# A newly discovered circovirus and its potential impact on human health and disease

**DOI:** 10.1097/JS9.0000000000001198

**Published:** 2024-02-16

**Authors:** Maria K. da Silva, Shopnil Akash, Jonas G.F. de Aquino, Shahina Akter, Umberto L. Fulco, Jonas I.N. Oliveira

**Affiliations:** a Federal University of Rio Grande do Norte, Natal, Brazil; b Daffodil International University; c Bangladesh Council of Scientific and Industrial Research (BCSIR), Dhaka, Bangladesh

Highlights

- A novel virus from the *Circoviridae* family and *Circovirus* genus was discovered in a patient in France with acute hepatitis of unknown etiology.
- A capsid (cap) sequence protein with 100% homology was found when compared with genome data provisionally called human circovirus type 1 (HCirV-1).
- Sequence comparison of the cap revealed amino acid homologies of 34.59% when compared with the reference sequence of porcine circovirus 3 (PCV3).
- Analyses revealed TCD8^+^ and TCD4^+^ epitopes with the highest combined antigenicity and immunogenicity scores, suggesting that these mutations may play an important role in replication and pathogenesis.

The recent discovery of an unidentified virus within the *Circoviridae* family and *Circovirus* genus in a French hepatitis patient marks a pivotal advancement in virology in addition, related circovirus designated as Human Circovirus 2 was identified in the blood of 2 intravenous drug users in China^1^. This finding transcends mere academic interest, signifying a matter of global health concern. It heralds a new era in comprehending circovirus diversity and their potential human infectivity, alongside the health implications of such infections.

Circoviruses, small single-stranded DNA viruses, mainly infect animals, including birds, pigs, and fish^2^. These viruses normally do not infect humans, but this view was challenged in a recent publication which identified a novel circovirus named human circovirus type 1 (HCirV-1) in a patient with acute hepatitis using shotgun metagenomics (SMG) and polymerase chain reaction (PCR)^1^. This virus has the same features as its conspecifics, including a replicase gene covering six conserved regions and a gene for the capsid protein, but it also has a unique ORF3 of undetermined function. In the context of the far-reaching impact of COVID-19, the significance of the discovery of HCirV-1 cannot be neglected^3^.

The occurrence of animal diseases has become a major health problem worldwide. According to a report by the Food and Agriculture Organization of the United Nations (FAO), an incredible 70% of diseases that have occurred since the 1940s can be attributed to animals^4^. This includes the current challenge posed by the novel coronavirus^5^. These alarming statistics highlight the need to raise awareness and take action to prevent the spread of these diseases^6,7^. Vaccination is an important strategy to prevent these diseases, and immunoinformatics promises to revolutionize the field of vaccine development^8–13^.

Recent studies on circoviruses have begun to shed light on their potential impact on human health^14^. PCV2 has been shown to infect and replicate in human cells in culture^14^. Studies of the effects of porcine circovirus type 1 on human blood leukocytes suggest possible interactions with the human immune system, potentially leading to altered immune responses or increased susceptibility to other diseases^15^. In addition, concerns regarding contamination of human vaccines with circoviruses highlight the indirect public health and safety implications of the virus and emphasize the need for strict safety protocols in vaccine production^16^. However, there is currently little direct evidence that circoviruses cause disease in humans; existing research emphasizes the importance of considering these viruses in the context of human health, including their role in immunomodulation, vaccine safety, and prevalence in the general population.

We compared the HCirV-1 cap sequence (Accession ID: WCO04044.1) with that of the next known circovirus, porcine circovirus 3 (PCV3) (Accession ID: YP_009315911), using MUSCLE^17^. These sequences were retrieved from the National Center for Biotechnology Information (NCBI). Sequence comparison of the cap revealed an amino acid homology of 34.59% compared to the reference sequence of PCV3.

A total of 138 amino acid mutations were detected in the cap protein of the analyzed sequence. These mutations occurred at different positions along the sequence and were mainly located in the regions of amino acid residues 2–10, 12–20, 24–41, 63–80, 85–99, 100–110, 145–157, 158–162, 163–171, and 196–214. To refine our analysis of these residues and prioritize the selection of epitopes, we used *in silico* approaches. We used peptide MHC binding affinity (MHC classes I and II) and servers to assess antigenicity and immunogenicity.

We used tools such as NetMHCIpan and NetCTL, which contain databases of experimental data from over 850 000 quantitative binding affinities (BAs) and mass spectrometry-eluted ligand (EL) peptides combined with 886 known MHC class I ligands. We also used NetMHCIIpan with an extensive data set of over 500 000 BAs and mass spectrometry-EL measurements. In addition, we used Vaxijen and IEDB immunogenicity for TCD8 and TCD4 epitopes, with a data set consisting of 100 known antigens and 100 non-antigens, and a database containing 1 590 097 peptide epitopes^14,15^. All raw data generated and analyzed are available in a scientific repository – 10.6084/m9.figshare.24749229.

Our analysis revealed that epitopes TCD8^+^ and TCD4^+^ had the highest combined values for antigenicity and immunogenicity (Fig. 1). Interestingly, the epitopes with the highest predicted antigenic potential were those that were most mutated, namely C^2–10^, C^102–110^, and C^165–173^ epitopes of TCD8. The TCD4 epitopes with the highest levels were C^45–59^, C^46–60^, C^47–61^, C^100–114^, and C^99–115^, suggesting that these mutations may play an important role in replication and pathogenesis.

**Figure 1:**
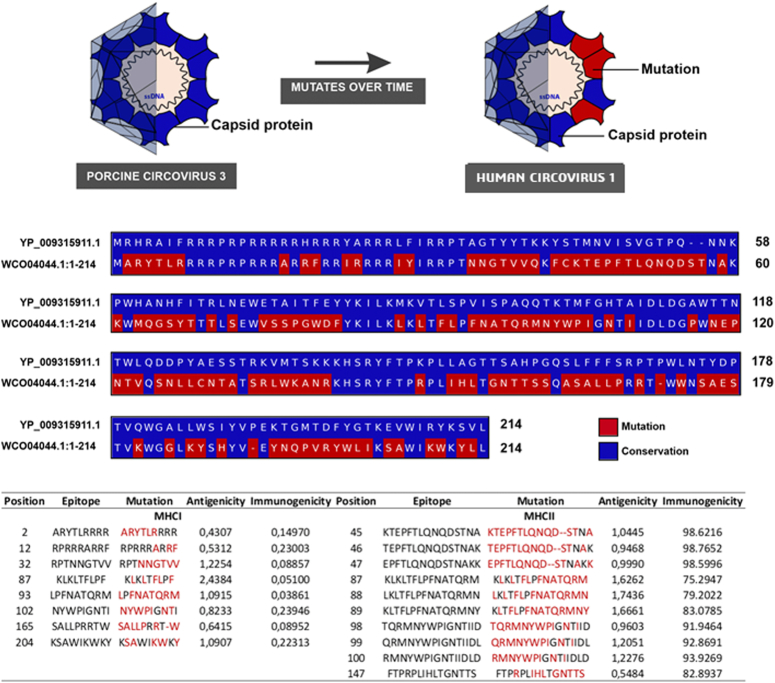
Matrix showing the amino acid sequence alignment of the capsid protein of porcine circovirus 3 (PCV3) and human circovirus 1. Conserved residues are shown in blue, and mutated residues in red. The table below the matrix shows the antigenicity and immunogenicity values of the predicted cytotoxic T cells (CTLs) and T helper cells (HTLs) epitopes of the HCirV-1 sequence.

The increasing threat to human health from circoviruses makes it necessary to identify vulnerable populations and develop effective vaccines and therapeutic measures. Immunocompromised individuals, particularly those with liver diseases such as hepatitis, are at increased risk of circovirus infection. This increased susceptibility was highlighted in a recent case study where a hepatitis patient was found to be infected with a circovirus, further highlighting the vulnerability of this patient group. Such observations underscore the urgent need for comprehensive research into viruses, including circoviruses, that are prone to infect humans to better understand their transmission dynamics and pathogenesis. This integration of technology is crucial in understanding and responding to emerging threats like circoviruses^18,19^.

The exploration of antiviral treatments, especially those targeting DNA viruses, is equally crucial. The study by Wongchanapai *et al*.^20^ on the comparative efficacy of chimeric porcine circovirus vaccines illuminates potential avenues for mitigating the impact of circoviruses. Furthermore, the vaccine development models for virus-induced diseases provide critical insights for creating effective strategies against circoviruses^9–12^.

Therefore, this information could be useful for experimental analyses of the relationship between HCirV-1 cap amino acid variations and strain pathogenicity and virulence. Further research is needed to better understand the impact of these mutations on the ability to infect humans. It is critical that we continue to monitor and research this and other emerging viruses to protect public health and prevent future outbreaks.

## Ethical approval

Not applicable/not required. This correspondent does not require any human/animal subjects to acquire such approval.

## Sources of funding

This research did not receive any specific grant from funding agencies in the public, commercial, or not-for-profit sectors.

## Author contribution

M.K.S., J.G.F.A., and S.A.^1^: conceptualization; M.K.S. and J.G. F.A.: investigation; M.K.S., S.A.^1^, S.A.^2^, and J.I.N.O.: formal analysis; M.K.S., J.G.F.A., and J.I.N.O.: methodology; J.I.N.O.: project administration; U.L.F. and J.I.N.O.: supervision; U.L.F., and J.I.N.O.: validation; M.K.S., J.G.F.A., S.A.^1^, and S.A.^2^: writing – original draft; U.L.F., and J.I.N.O.: writing – review and editing.

## Conflicts of interest disclosure

The authors have no conflicts of interest to declare that are relevant to the content of this article.

## Research registration unique identifying number (UIN)

1. Name of the registry: not required.
2. Unique identifying number or registration ID: not required.
3. Hyperlink to your specific registration (must be publicly accessible and will be checked): not required.

## Guarantor

I, Jonas I.N. Oliveira, take full responsibility for the work and/or the conduct of the study, had access to the data, and controlled the decision to publish.

## Data availability statement

All data used to support the findings of this study are included in the article.
